# Molecular Epidemiology and Genotyping of Hepatitis B Virus of HBsAg-Positive Patients in Oman

**DOI:** 10.1371/journal.pone.0097759

**Published:** 2014-05-16

**Authors:** Said Ali Al Baqlani, Bui Tien Sy, Boris A. Ratsch, Khalid Al Naamani, Salah Al Awaidy, Suleiman Al Busaidy, Georg Pauli, C.-Thomas Bock

**Affiliations:** 1 Central Public Health Laboratory – Ministry of Health, Muscat, Oman; 2 Department of Infectious Diseases, Robert Koch Institute, Berlin, Germany; 3 Vietnam Military Medical University, Ha Dong, Ha Noi, Viet Nam; 4 Institute of Tropical Medicine, University of Tübingen, Tübingen, Germany; 5 Armed Forces Hospital – Ministry of Defense, Muscat, Oman; 6 Office of the Undersecretary for Health Affairs – Ministry of Health, Muscat, Oman; University of Cincinnati College of Medicine, United States of America

## Abstract

**Background:**

Hepatitis B virus (HBV) infection is a major global health burden with distinct geographic public health significance. Oman is a country with intermediate HBV carrier prevalence; however, little is known about the incidence of HBV variants in circulation. We investigated the HBV genotype distribution, the occurrence of antiviral resistance, and HBV surface antigen (HBsAg) escape mutations in HBsAg-positive patients in Oman.

**Methods:**

Serum samples were collected from 179 chronically HBV-infected patients enrolled in various gastroenterology clinics in Oman. HBV genotypes were determined by sequencing and phylogenetic analysis. Mutations in the HBV polymerase and the HBsAg gene were characterized by mutational analysis.

**Results:**

HBV genotypes D (130/170; 76.47%) and A (32/170; 18.28%) are predominant in Oman. The HBV genotypes C and E were less frequent (each 1.18%), while the HBV genotypes B, G, F, and H were not detected. Four patients revealed HBV genotype mixtures (HBV-A/D and D/C). The analyses of vaccine escape mutations yield that 148/170 (87.06%) HBV sequences were wild type. 22/170 (12.94%) HBV sequences showed mutations in the “a” determinant of the HBsAg domain. Two patients showed the described HBV vaccine escape mutation sP120T. 8/146 (5.48%) HBV isolates harbored mutations in the HBV polymerase known to confer resistance against antiviral therapy. Especially the lamivudine resistance mutations rtL180M/rtM204V and rtM204I were detected.

**Conclusion:**

This study shows the distribution of HBV genotypes, therapy resistance, and vaccine escape mutations in HBV-infected patients in Oman. Our findings will have a major impact on therapy management and diagnostics of chronic HBV infections in Oman to control HBV infection in this intermediate HBV-endemic country.

## Introduction

Despite the introduction of a safe and effective vaccine against hepatitis B virus (HBV) in 1982, hepatitis B remains a global public health burden resulting in more than 600,000 deaths worldwide per year [1]. Clinical manifestations of HBV infection range from inapparent infection to fulminant hepatic failure. Chronic infection develops in approximately 5% of immunocompetent HBV-infected adults, but up to 100% of infected newborns may become HBV carriers. The long-term consequences of chronic HBV infection include liver cirrhosis and hepatocellular carcinoma (HCC). These life-threatening liver disease complications can affect 15%–40% of HBV carriers who acquired the virus early in life [2], [3].

Eight HBV genotypes (A–H) have been described based on nucleotide divergence over the entire genome sequence of more than 8% [4], [5]. HBV genotypes have distinct geographic distribution, with genotype A found predominantly in Northwest Europe, North America, and Central and sub-Saharan Africa; genotypes B and C in Southeast Asia, China, and Japan; genotype D in the Mediterranean, the Middle and Far East, and India; genotype E in Africa; genotype F in Native Americans, Polynesia, and Central and South America; genotype G in the United States and France; and genotype H in Central America [5]–[7]. Africa is one of the highly endemic regions for HBV, with five HBV genotypes (A–E) predominating [8]. HBV genotypes show not only distinct geographic distribution but even within regions prove to be an invaluable tool in tracing the molecular evolution, patterns, and mode of spread of HBV [9]. The natural history of chronic hepatitis B (CHB) differs between HBV genotypes with regard to progression to liver fibrosis and development of HCC [10]–[14]. In addition, HBV genotypes differ in their response to antiviral treatment, e.g. susceptibility to interferon-alpha is greater in HBV genotype A-infected patients than in those infected with genotypes D, B, and C [15]. In contrast, the response to treatment with nucleoside/nucleotide analogues is rather independent of HBV genotypes [16], [17] and can possibly influence vaccination efficacy against HBV [18].

Oman is a country with an intermediate prevalence of HBV carriers (2.8–7.1%) [19], [20]. According to a retrospective study conducted in 2010 using serum samples collected for the World Health Survey, it was observed that the prevalence of HBV infection in the Omani population over all age groups was 5.8% (unpublished data). In 1990, Oman implemented vaccination of all newborns according to the WHO recommendation [1]. The impact on vaccination efficacy and coverage was evaluated in 2005 in a nationwide survey, showing that 15 years after introduction of HBV vaccination of newborns the prevalence of CHB in children dropped from 2.3% in 1990 to 0.5% in 2005 [21].

Little is known about HBV genetic diversity including genotype distribution, the prevalence of antiviral resistance, and surface antigen vaccine escape mutations in circulation in Oman. Therefore, we determined the prevalence of HBV genotypes among individuals who have been tested positive for HBsAg. Furthermore, we explored the prevalence of “a” determinant vaccine escape mutants and antiviral treatment resistance mutations.

## Materials and Methods

### Study Subjects

One hundred seventy-nine chronically HBV-infected patients were included in this study. All HBsAg-positive patients were out-patients and were enrolled at three tertiary hospitals, the Royal Hospital, Khoula Hospital, and Armed Forces Hospital in Muscat and additionally at eleven regional hospitals (Sohar H., Suweiq H., Rustaq H. [all in Batinah]; Buraimi H. [Buraimi]; Nizwa H., [Al Dhakhliya]; Ibri H. [Dhahira]; Salalah H. [Dhofar]; Khasab H. [Musandam]; Ibra H., Sur H. [both in Sharqiya]; and Haima H. [Wosta]) to represent the whole Sultanate of Oman. Since the patient samples of this study were obtained from out-patients, no information on treatment history was available. All patients were confirmed positive for HBsAg and negative for anti-HCV and anti-HDV. Serum samples were collected from the HBsAg-positive patients and stored at −80°C until use.

### Ethical Approval

The study was approved by the ethical committee of the Ministry of Health, Oman. Informed written consent was given by all participants.

### Serological Markers

Serological markers for HBV profiles for HBV antigens (HBsAg, HBeAg) and antibodies (anti-HBc IgM, anti-HBc IgG, and anti-HBe) were determined using commercially available one-step enzyme immunoassay kits (Monolisa, Bio-Rad, Hercules, CA, USA), Anti-HCV was determined using Murex version 4.0 (Abbott Laboratories, Abbott Park, IL, USA) and anti-HDV IgG/IgM using ELISA assays (DRG International, Springfield, NJ, USA). All serological approaches were carried out following the manufacturers’ instructions.

### Nucleic Acid Extraction

Nucleic acid was extracted from 200 µl of patient sera using the QIAamp DNA extraction kit (Qiagen, Hilden, Germany) following the manufacturer’s instructions, subsequently diluted in 10 µl aliquots and stored at −80°C until use.

### Detection of HBV Genomes

The presence of HBV genomes was determined using HBV-specific nested PCR as described previously [22] with minor modifications. HBV primer sequences are described in Table 1. In brief, the first PCR was performed using sense primer HBV-022 and antisense primers HBV-065 and HBV-066. The nested PCR was carried out using primer HBV-024 and antisense primers HBV-041 and HBV-064 which amplified a 332 bp fragment spanning from nt455 to nt786 (numbering according GenBank accession number HM011485). PCR was performed with 5 µl of isolated nucleic acid, 200 nM of each primer and the HotStarTaq Master Mix Kit (Qiagen, Hilden, Germany). Thermal cycling parameters were initial denaturation at 94°C for 15 min, followed by 35 cycles of 30 sec at 94°C denaturation, 30 sec at 52°C annealing, and 45 sec at 72°C extension, followed by a final extension of 5 min at 72°C. Cycling parameters for the second PCR remained the same as in the first one except that the number of cycles was increased to 40. PCR products were analyzed on 1.5% tris-borate-EDTA (TBE) agarose gels. Serum HBV DNA from a patient with a high HBV titer diluted into 1.000 copies/5 µl as positive control (generous gift from Prof. Wedemeyer, Hannover Medical School, Hannover, Germany) and an HBV-negative control were included in any run. The nested PCR was evaluated with HBV QCMD panels HBVDNA11A and HBVGT11 for HBV detection and genotyping, respectively (Quality Control for Molecular Diagnostics, www.qcmd.org). Each sample was tested at least in duplicate. Sample processing (DNA/RNA extraction, template preparation, and master-mix preparation) and PCR were done in separate laboratory rooms which are all certified for molecular diagnostics using standard precautions to prevent assay contamination.

**Table 1:** HBV-specific primers for detection, genotyping, and mutational analysis.

N°	Name	Sequence 5′-3′	Position
1.	HBV-022	TGCTGCTATGCCTCATCTTC	414–433
2.	HBV-065	CAAAGACAAAAGAAAATTGG	822–803
3.	HBV-066	CACAGATAACAAAAAATTGG	822–803
4.	HBV-024	CAAGGTATGTTGCCCGTTTGTCCT	455–478
5.	HBV-041	GGACTCAMGATGYTGTACAG	786–767
6.	HBV-064	GGACTCAMGATGYTGCACAG	786–767
7.	HBV-069	CCTGCTGGTGGCTCCAGTTC	56–75
8.	HBV-070	CGCAGTATGGATCGGCAGAGG	1275–1255
9.	HBV-001	CTGCTGGTGGCTCCAGTTCAGGA	57–79
10.	HBV-017	GGGGTTGCGTCAGCAAACACT	1199–1179
11.	HBV-025	GGTATAAAGGGACTCACGATG	795–775

Nucleotide position is according to the HBV reference strain GenBank accession number HM011485.

### Identification of HBV Genotypes and Genotype Mixtures

**Figure 1:**
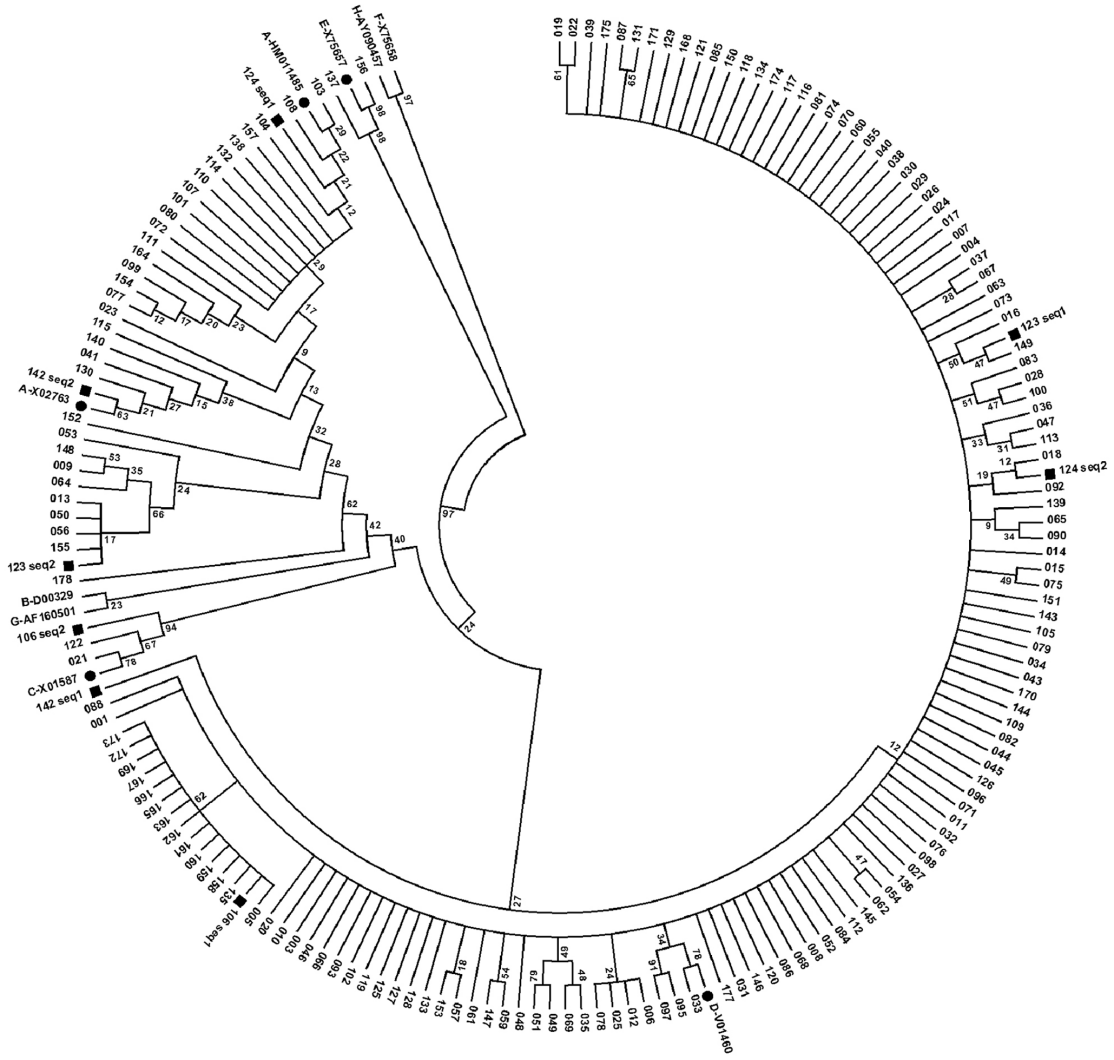
Phylogenetic analysis of Omani HBV strains. Phylogenetic analysis inferred from distance analysis (Kimura 2 parameters model) and neighbor-joining reconstruction from a partially overlapping region of P and S genes (from nt479 to nt766) of 170 Omani HBV DNA-positive isolates. The reference strains were denoted by HBV genotype and “•” signal with the GenBank accession number, respectively (for example: “A-HM011485•”). The Omani samples were numbered, and especially the double-infected samples were highlighted by the “▪” signal. (The numbers at the nodes indicate bootstrapping values.).

HBV genotypes were determined by direct sequencing of HBV-specific amplicons of the HBV S gene obtained by the HBV detection PCR. Nested PCR products were purified using the Exo SAP-IT kit (USB Corporation, Cleveland, OH, USA) according to the manufacturer’s instructions. Sequencing reactions were performed using 1–5 µl of purified PCR products, 1 µl of BigDye reaction mix (Life Technologies/Applied Biosystems, Darmstadt, Germany), and 0.5 µM of primers HBV-024 and HBV-025, respectively. Obtained sequences were manually edited and analyzed using BioEdit 9.7 (http://www.mbio.ncsu.edu/bioedit/bioedit.html) and Geneious Pro software (Version R6, Biomatters Ltd, Auckland, New Zealand). For genotyping, phylogenetic analysis with corresponding sequences of nine HBV reference sequences, representing the eight HBV genotypes A to G, was performed using the MEGA 5 software [23]. The molecular evolution was inferred by using the Neighbor-Joining method with 1,000 bootstrap replicates. Evolutionary distances were computed using the Maximum Composite Likelihood method [23]. For independent confirmation, the genotyping online tool at http://www.hepseq.org was utilized. HBV reference strains in GenBank were HBV-A: HM011485, X02763; HBV-B: D00329; HBV-C: X01587; HBV-D: V01460; HBV-E: X75657; HBV-F: X75658; HBV-G: AF16050; and HBV-H: AY090457 (Fig. 1).

HBV genotype mixtures were discovered by aligning sequences from the genotyping PCR with sequences from the mutational analysis. Obvious sequence differences, not explainable as occurrence of quasi-species, were analyzed separately and confirmed by sub-cloning and sequencing. Sub-cloning was performed using TOPO TA Cloning Kit and Top10 competent cells (Life Technologies/Applied Biosystems, Darmstadt, Germany) following the manufacturer’s instructions. At least 10 clones were sequenced for identifying HBV genotype mixtures.

In order to identify possible HBV genotype recombination, all HBV sequences were analysed using the Recombination Detection Program (version 4.22) as described recently [24].

### Determination of Antiviral HBV Resistance and HBV Vaccine Escape Mutations

**Figure 2:**
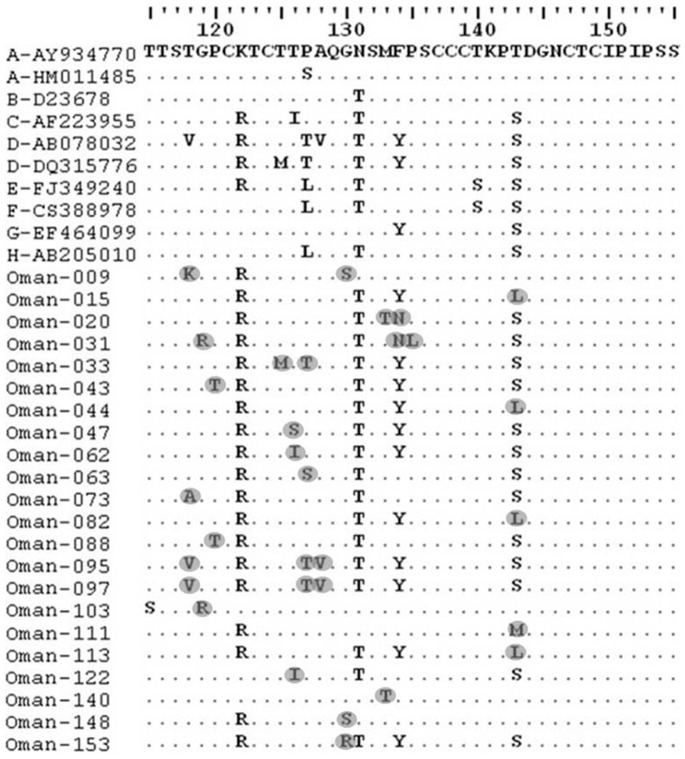
Analysis of escape mutants of Omani HBV isolates. The amino acid sequences in the “a” determinant region of the Omani escape mutant isolates were aligned with the corresponding region of the reference sequences (HBV-genotypes A to H; GenBank accession numbers are denoted) by ClustalW with Neighbor Joining method and 1000 bootstrap replicates. Amino acid substitutions were highlighted.

**Figure 3:**
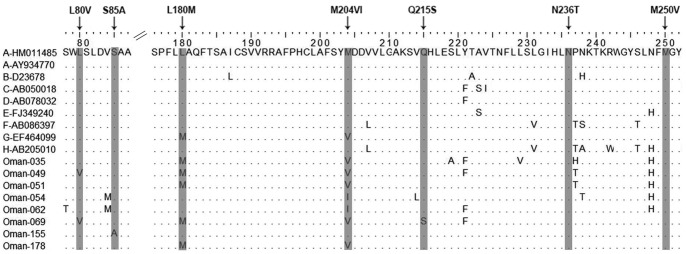
Analysis of antiviral therapy resistant mutations of Omani HBV isolates. The amino acid sequences of the HBV polymerase (reverse transcriptase region; aa rtM1 to rtL275) of the Omani resistant mutation isolates were aligned with the corresponding region of the reference sequences (HBV-genotypes A to H; GenBank accession numbers are denoted) by ClustalW with Neighbor Joining method and 1000 bootstrap replicates. Described antiviral resistant mutations were highlighted and denoted at the top (e.g., L80V).

Presence of HBV antiviral therapy resistance mutations and HBV vaccine escape mutations was determined by established in-house PCR and subsequent sequencing. In brief, in the first PCR round the HBV consensus primer pair HBV-069 and HBV-070 was used. For the nested HBV PCR sense primer HBV-001 and antisense primer HBV-017 were used for amplifying a 1143 bp fragment spanning from nt57 to nt1199 (numbering according to HM011485). Amplified fragments were purified using the Exo SAP-IT kit (USB Corporation, Cleveland, OH, USA) and sequenced using primers HBV-001, HBV-025, and HBV-017. Fully overlapping sequences from primers HBV-001 and HBV-025 were used to discover escape mutations, while sequences obtained with primers HBV-001 and HBV-017 were analyzed to determine antiviral resistance. Sequences were manually edited, aligned with prototype reference sequences (accession numbers for the HBV prototypes are shown in Fig. 2 and 3), and translated into amino acid sequences using the Geneious Pro software (Version R6, Biomatters Ltd) to identify amino acid substitutions. All primer sequences are listed in Table 1.

The 288 bp fragment located between nt479 and nt766 coding for amino acid (aa) 109–204 of the HBsAg and the 858 bp fragment between nt208 and nt1065 coding for aa 27–286 of the reverse transcriptase (rt) domain of the HBV polymerase were used for the analysis of drug resistance mutations and vaccine escape mutations, respectively. 146 sequences of this analysis were submitted to GenBank (accession numbers: KJ585434–KJ585579).

### Statistical Analysis

Statistical analysis was performed using SPSS release 20 (IBM Corporation, Armonk, NY, USA) and Prism5 software (version 5.01, GraphPad Software, San Diego, CA, USA, www.graphpad.com). Categorical variables were compared by Fisher’s exact test. Continuous quantitative variables were compared by using the Mann-Whitney U test, with a 2-tailed p-value <0.05 considered to be statistically significant.

## Results

### Baseline Characteristics of the HBsAg-positive Omani Patients

One hundred seventy-nine HBsAg-positive out-patients from Oman were included in this study. 54/179 (30.17%) of the patients were female and 124/179 (69.27%) were male; for one patient no gender information was available. The median age of patients was 35 years (range: 18–60 years). Median serum HBV loads of the patients ranged from 1.08 log_10_ to 7.04 log_10_ HBV IU/ml. The baseline characteristics of the patients are summarized in Table 2.

**Table 2:** Clinical profiles of HBsAg-positive patients of Oman.

Variables	Values
Median age (y)	35y (18–61 years)
Sex (male/female)	124/54
HBV load (log_10_ IU/ml)	1.08 to 7.04
HBsAg positive	179/179 (100%)
HBeAg positive	7/179 (3.91%)
HBeAg negative	120/179 (67.04%)
anti-HBeAg positive	120/179 (67.04%)
HBeAg and anti-HBeAg negative	4/179 (2.23%)

### Prevalence and Geographic Distribution of HBV Genotypes in Oman

Serum samples from 179 HBsAg-positive patients were collected in all nine governorates at 14 gastroenterologic clinics, representative for the whole Sultanate of Oman according to its population density with approximately 90% of inhabitants living in the North. 146 (81.6%) samples were from the northern and 33 (18.4%) samples from the southern part of Oman.

HBV genomes were detected by PCR in 170 samples (137 in the northern and 33 in the southern regions). Sequencing of PCR products and subsequent phylogenetic analysis were performed to determine HBV genotypes. Of all circulating HBV genotypes, genotypes D (130/170; 76.47%) and A (32/170; 18.28%) are predominant in Oman. HBV genotypes C (2/170; 1.18%) and E (2/170; 1.18%) were less frequent and the HBV genotypes B, F, G, and H were not detected (Fig. 1). Noteworthy, four patients (2.35%) showed HBV genotype mixtures implying double infections (patient numbers 106, 123, 124, and 142). Three of these patients were infected with HBV genotypes A and D, and patient #106 was infected with HBV genotypes C and D (Fig. 1). No HBV genotype recombination could be detected in the analysed patient samples.

Comparison of the HBV genotype distribution between the North and the South of Oman revealed only minor differences. In the northern region the prevailing HBV genotype was D (107/137; 78.10%), followed by genotype A (23/137; 16.79%), including 3/137 (2.19%) HBV D/A genotype mixtures, and two (1.46%) of each HBV genotypes C and E. HBV genotypes D (23/33; 69.70%) and A (9/33; 27.27%) were also the prevalent HBV genotypes in the South of Oman, and only one isolate (3.03%) represented HBV genotype mixtures (HBV D/C).

### Prevalence of HBV Vaccine Escape Mutations in HBsAg-positive Omani Patients

In order to determine the prevalence of HBV vaccine escape mutations circulating in Oman, mutational analysis was performed on the 170 HBV isolates. Major vaccine escape mutations have been described to be located in the “a” determinant of the small surface protein (SHBsAg, aa 124–147). We therefore analyzed the HBV sequences of the surface gene of the HBV genome from nt497 to nt635, coding for aa115 to aa160 of SHBsAg (numbering is according to GenBank accession number HM011485). Of the170 HBV isolates, 148/170 (87.06%) showed wild-type sequences, while 22/170 (12.94%) HBV surface antigen sequences showed mutations in the HBsAg gene. In particular, mutations at aa positions 118–120, 125–128, 130, 133–135, and 143 were observed. Sixteen patients showed only one mutation, and three patients showed two and three mutations, respectively, in the “a” determinant region (Fig. 2 and Table 3).

**Table 3:** Mutations in the “a” determinant domain of Omani HBV isolates.

Samples	T118K/A/V	G119R	P120T	T125M	T126I/S	P127T/S	A128V	G130S/R/N	M133T	Y/F134N	P135L	T/S143L/M
**OMAN-009**	+							+				
**OMAN-015**												+
**OMAN-020**									+	+		
**OMAN-031**		+								+	+	
**OMAN-033**				+		+						
**OMAN-043**			+									
**OMAN-044**												+
**OMAN-047**					+							
**OMAN-062**					+							
**OMAN-063**						+						
**OMAN-073**	+											
**OMAN-082**												+
**OMAN-088**			+									
**OMAN-095**	+					+	+					
**OMAN-097**	+					+	+					
**OMAN-103**		+										
**OMAN-111**												+
**OMAN-113**												+
**OMAN-122**					+							
**OMAN-140**									+			
**OMAN-148**								+				
**OMAN-153**								+				

The classical diagnostic/vaccine escape mutations, G145R and D144E, could not be detected. However, the well-described vaccine escape mutation P120T was found in two sequences of HBsAg-positive patients (samples 43 and 88) while both of them could be related to HBV genotype D (Fig. 2 and Table 3). Other possible vaccine escape mutations were detected at positions T126 (T126S) and M133 (M133T); however, their impact on vaccine escape is under discussion. Further identified S-gene mutations are not associated with diagnostic and vaccine escape and were S-gene variants.

The distribution of HBV escape mutations of HBV isolates from different regions of Oman, gender, HBV genotypes, the status of HBeAg and anti-HBe, age, and HBV load is displayed in table 4. No correlation was found for geographic distribution, gender, HBV genotype, status of HBeAg and anti-HBe, age and HBV load of escape mutations detected in the HBsAg-positive samples from the north compared to the south of Oman (p>0.5).

**Table 4:** Distribution of S-gene variants of Omani HBV isolates.

		No mutation			S-gene mutations		
		Count	Mean	Median	Count	Mean	Median
Region	North	118	–	–	19	–	–
	South	30	–	–	3	–	–
Gender	F	46	–	–	6	–	–
	M	102	–	–	16	–	–
Age		148	37	33	22	44	41
HBeAg	ND	3	–	–	1	–	–
	Neg	72	–	–	9	–	–
	Pos	73	–	–	12	–	–
Anti-HBe	ND	3	–	–	1	–	–
	Neg	77	–	–	12	–	–
	Pos	68	–	–	9	–	–
HBV Genotype	A	27	–	–	5	–	–
	C	1	–	–	1	–	–
	D	114	–	–	16	–	–
	D-A	3	–	–	0	–	–
	D-C	1	–	–	0	–	–
	E	2	–	–	0	–	–
Viral load^a^		148	4,55	4,56	22	4,03	3,52

a Values are given as log_10_ international units (IU)/ml; ND: Not done; F = female, M = male.

### Prevalence of HBV Resistance Mutations to Antiviral Therapy

Chronically HBV-infected patients in Oman were treated with nucleotide/nucleoside analogues (NA) according to European/American guidelines. To identify the antiviral therapy resistance mutations, the obtained Omani patient sequences located between nt130 to nt954 coding aa rtM1 to rtL275 (numbering is according to GenBank accession number HM011485) were aligned with the corresponding regions of reference sequences and subsequently translated into aa sequences. Of 170 HBV DNA-positive samples, 146 were successfully sequenced using the primer pair HBV-001 and HBV-017. Mutational analysis showed that eight of the 146 HBsAg-positive patients (5.48%) exhibit NA resistances. Among them seven patients had lamivudine resistance mutations (LAM) and one had the adefovir resistance mutation (ADV; S85A of patient #155) (Fig. 3). Notably, one patient (patient #069) showed four mutations (L80V, L180M, M204V, and Q215S) and patient #049 three mutations (L80V, L180M, and M204V). The N236T adefovir dipivoxil resistance mutation and other previously described resistance mutations against other antiviral drugs (e.g. entecavir, telbivudine) could not be detected in the HBsAg-positive patients.

The distribution of antiviral resistance mutations of HBV isolates from different regions of Oman, gender, HBV genotypes, the status of HBeAg and anti-HBe, age, and HBV load were analyzed and are displayed in table 5. A correlation could be identified showing that drug resistance mutations were significantly more abundant in anti-HBe-negative individuals (one tailed Fisher’s exact test, p = 0.045). However, no correlation could be detected for geographic distribution, gender, HBV genotype, HBeAg status, age, and viral load (p>0.5).

**Table 5:** Distribution of antiviral resistance mutations.

		No mutation			Resistance mutations		
		Count	Mean	Median	Count	Mean	Median
Region	North	111	–	–	6	–	–
	South	27	–	–	2	–	–
Gender	F	40	–	–	3	–	–
	M	98	–	–	5	–	–
Age		138	37	33	8	37	34
HBeAg	ND	2	–	–	0	–	–
	Neg	69	–	–	2	–	–
	Pos	67	–	–	6	–	–
Anti-HBe*	ND	2	–	–	0	–	–
	Neg	69	–	–	7	–	–
	Pos	67	–	–	1	–	–
Genotype	A	28	–	–	2	–	–
	C	2	–	–	0	–	–
	D	107	–	–	6	–	–
	E	1	–	–	0	–	–
Viral load^a^		138	4,50	4,43	8	5,44	5,90

*denotes p<0.05; ^a^Values are given as log_10_ international units (IU)/ml; ND: Not done; F = female, M = male.

## Discussion

The prevalence of HBV infection varies to a great extent in different areas and countries, depending on a number of factors that include implementation of the HBV vaccination program, screening of blood samples from blood donors, or education of the population about the risk factors for HBV infection. Oman, a country with intermediate HBV carrier prevalence of 2–7%, has implemented HBV vaccination of all newborns in the country for the local population and immigrants free of charge in 1990. However, little is known about the prevalence of HBV genotypes, HBV resistance mutants to antiviral drugs, and HBV vaccine escape mutants in circulation in Oman.

Increasing evidence supports the view that different HBV genotypes have a significant role in determining the clinical outcome of liver diseases and the response to antiviral therapies [14], [25]–[27]. Accordingly, in this study we have analyzed the geographic distribution of HBV genotypes and the prevalence of vaccine escape and antiviral treatment resistance mutants of 179 HBsAg-positive patients from 14 gastroenterology clinics of geographically distinct regions of Oman. All patients were out-patients and were enrolled in hospitals throughout Oman. This patient cohort might be representative of the general HBsAg-positive population of Oman. Of 179 HBsAg-positive patients, HBV DNA was detected in 170 patients (94.97%). Molecular-epidemiologic analysis enabled the detection of four of the eight HBV genotypes, namely genotypes A, C, D, and E, with a predominance of genotypes A (18.28%) and D (76.47%). This is in accordance with previous studies of HBV genotype distribution in other regions of the Middle East and neighboring countries or those in close contact to Oman. HBV genotype D is predominant in neighboring countries Saudi Arabia (81.4%) and the Republic of Yemen (90%), but also prevalent in Egypt, Turkey, Iran, and Tunisia [28]–[33].

Few studies have reported the occurrence of mixed HBV genotype infections and the effect of double infections on the clinical outcome or on viral replication [14], [34]. We found HBV genotype mixtures in 4 out of 170 (2.35%) serum samples, in particular three with HBV-A/D and one with D/C. Notably, no HBV genotype recombination could be detected in the Omani HBV isolates. The prevalence of mixed HBV genotype (2.35%) found in Oman was low when compared to other studies from Asia, where 10.6% (Eastern China) [35] and 16.9% (Vietnam) [14] of the hepatitis B patients revealed HBV genotype mixtures. This may be due to the low prevalence of HBV in this country (2%–7%) in contrast to regions in Asia with a high prevalence, like China with about 10% [36] and Vietnam with up to 20% [37] of HBsAg-positive individuals. However, the high vaccination coverage might also have had an impact on the infection pattern observed in Oman.

The common hepatitis B vaccines contain the major HBsAg protein that induces immune response to the major hydrophilic region (MHR), the so called “a” determinant which maps to aa residues 100–160 of the HBV surface protein [38]. Various vaccine/immune and diagnostic escape mutations have been described which are detectable mainly in the most antigenic MHR located in the region of aa120–aa147 [39]–[44]. The best-described and stable vaccine escape mutations are the sG145R, sD144E/A, and sP120T mutations [45], [46]. HBsAg mutations in the “a” determinant can lead to conformational changes with altered antigenicity and failure to neutralize the virus, to escape diagnostic assays, and are associated with liver disease progression like fulminant hepatitis, cirrhosis, and HCC [43], [46]–[48]. The occurrence of HBsAg mutations is mainly caused by vaccine and/or hepatitis B immunoglobulin (HBIG) administration, natural selection by the host immune response, and antiviral therapy pressure inducing antiviral resistance mutants which alter simultaneously the amino acid composition of the surface antigen [49]–[51]. Of the 170 HBV isolates analyzed, 148 (87.06%) showed wild-type sequences and only 22 (12.94%) revealed mutations in the HBsAg gene and of these two isolates (1.1%) showed the described vaccine escape mutation sP120T. Other vaccine escape mutations, especially the G145R and D144E/A mutation, could not been detected. Our result is in good agreement with recent reports revealing a prevalence of HBsAg vaccine escape mutations of 0.7% to 28% depending on the region, age of HBV-infected individuals, and endemicity of HBV [42], [48], [52]. The low frequency of vaccine escape mutations implies that there is obviously no threat to the HBV vaccination program in Oman. However, a surveillance program to observe the situation of circulating HBV escape mutations in Oman is needed to determine the prevalence of HBV escape mutations more comprehensively. Most of the mutations detected in the “a” determinant of the HBV isolates from the patients of Oman were located in loop 1 (B cell epitope; aa118–120, 126–128, 130, and 133–135). Of these, the well-characterized sP120T mutation could be detected in 2/170 HBV-infected patients. The identified sT/S143L/M mutations of the HBsAg gene, mapped to loop 2 of the “a” determinant, and were thought to be associated with vaccination allowing ongoing replication of HBV [53]. However, in this domain (loop 2) the most prominent vaccine escape mutations sG145R and sD144E/A were not found in the patient collective. Mutations in the S-gene region between aa 120 and 145 have been discussed to affect the antigenic structure of the HBsAg. We found the sP120T, sT126S, sM133T, and sT/S143L/M mutations in the HBsAg-positive patients while these mutations have been previously described as possible vaccine escape mutations (reviewed in: [54]). However, these mutations were less frequent (4.1%). Other mutations detected in the S-gene of the patient isolates have not been described as escape mutations so far and therefore could be classified as “a” determinant variants.

Besides interferon-alpha, at present five nucleoside/nucleotide analogues (NAs) have been introduced worldwide for treatment of chronic hepatitis B (CHB) targeting the viral polymerase (reverse transcriptase, RT). Of these, lamivudine (LAM, 3TC) was the antiviral drug first approved in 1995. Although the introduction of NAs for therapy of CHB significantly reduces disease progression and efficiently controls HBV replication, unfortunately, NAs can select resistance mutations in the HBV RT-domain which is a major limiting factor for long-term viral suppression [55]. For LAM the prevalence of resistance mutations rapidly rises up to 80% after four years of treatment. The other NAs, adefovir dipivoxil, entecavir, telbivudine, and tenofovir disoproxil, show higher genetic barriers with a resistance development for tenofovir of 0% to 5.9% after treatment for three years [39]. Besides other previously described resistance mutations, the best-described drug resistance mutations are the LAM resistance mutations rtL180M and rtM204V/I and the adefovir resistance mutations rtA181V/T and rtN236T [39]. The LAM mutations rtM204V/I are mapped to the catalytic center of the viral RT domain, the YMDD motif. Although we had no information on hand how many and which ones of the 170 CHB patients of our study have been under anti-viral treatment and which NA therapy was used, we found 5.48% NA-resistance mutations, seven of them conferring resistance to LAM. One patient was determined to have the suspected adefovir-resistant mutation (rtS85A). Other antiviral therapy resistance mutations, such as rtA181V/T or rtN236T, were not detected. According to the guidelines on the management of chronic hepatitis B, in the case of resistance to LAM and also ADV the therapy should be switched or add-on to a nucleotide analogue treatment option like tenofovir disoproxil fumarate [56]. The relatively low rate of approx. 5.48% LAM-resistant patients is in agreement with a recent report from the US showing <2% in untreated CHB patients [57].

Further analysis showed that there was no statistical correlation between the geographic distribution, gender, age, HBV genotypes, status of HBeAg and anti-HBe, and HBV load of the vaccine escape and resistance mutations if comparing HBsAg-positive samples from the north with the south of Oman. However, a significant correlation between the anti-HBe status (anti-HBe negative) and the frequency of antiviral resistance mutations could be identified. This finding may suggest that anti-HBe-negative individuals might be more likely to develop resistance mutations. However, this finding has to be proven in further studies in more detail.

In conclusion, this study shows for the first time the prevalence and geographic distribution of HBV genotypes in Oman, with a predominance of HBV genotypes D and A. HBV genotype mixtures of A and D were also observed to a low extent. Mutational analysis of the HBV isolates revealed a low prevalence of vaccine escape mutants in the HBsAg-positive patients which is indicative of the effectivity of the HBV vaccination program in Oman. The incidence of antiviral therapy resistance mutations of the CHB patients was low; however, the identification of LAM and ADV resistance mutations recommend the adjustment of the therapy management to international guidelines. Our findings will therefore have major impact on the therapy management and diagnostics of CHB patients in Oman.
